# Spatial infectious disease epidemiology: on the cusp

**DOI:** 10.1186/s12916-018-1184-6

**Published:** 2018-10-18

**Authors:** G Chowell, R Rothenberg

**Affiliations:** Department of Population Health Sciences, School of Public Health, Georgia State University, Atlanta, GA USA

**Keywords:** Infectious diseases, Spatial methods, Cluster, Hotspot, Spatiotemporal modeling, Geospatial information

## Abstract

Infectious diseases continue to pose a significant public health burden despite the great progress achieved in their prevention and control over the last few decades. Our ability to disentangle the factors and mechanisms driving their propagation in space and time has dramatically advanced in recent years. The current era is rich in mathematical and computational tools and detailed geospatial information, including sociodemographic, geographic, and environmental data, which are essential to elucidate key drivers of infectious disease transmission from epidemiological and genetic data. Indeed, this paradigm shift was driven by dramatic advances in complex systems approaches along with substantial improvements in data availability and computational power. The burgeoning output of infectious disease spatial modeling suggests that we are close to a fully integrated approach for early epidemic detection and intervention. This special collection in *BMC Medicine* aims to bring together a broad range of quantitative investigations that improve our understanding of the spatiotemporal transmission dynamics of infectious diseases in order to mitigate their impact on the human population.

## Background

Infectious diseases, including respiratory (influenza, pneumonia, respiratory syncytial virus), vector-borne (malaria, dengue, chikungunya, and Zika), and sexually transmitted diseases (HIV, syphilis), continue to pose a significant public health burden despite the great progress made in their prevention and control over the last few decades. Fortunately, our understanding of mechanisms driving their propagation in space and time and their control has radically evolved from shamanistic to analytic. Modern quantitative computational tools and highly resolved geospatial demographic, epidemiological, and genomic data are enabling actionable insights for public health in near real time. Indeed, the rapid increase in data-driven spatial modeling output over the last three decades is connected to significant advances in complex system modeling approaches along with substantial improvements in data availability and computational capacity. For comparison, when the 1918–1919 influenza pandemic struck the globe, it was not known until years later that the influenza virus was responsible for the deaths of 20 to 100 million people. Additionally, in most parts of the world, morbidity records were scarce and the majority of vital records were archived in churches and cemeteries. Consequently, the geographic point-of-origin and spatiotemporal patterns of spread of this lethal virus remain poorly understood [1]. In contrast, genetic sequence data from patient samples during the 2009 A/H1N1 influenza pandemic or the recent 2014–2016 Ebola epidemic in West Africa allowed researchers to reconstruct geographic transmission patterns and monitor their spread with reasonable precision [2–4].

This special collection in *BMC Medicine* aims to bring together a broad range of quantitative investigations that generate actionable results on the spatiotemporal transmission dynamics of infectious diseases. These contributions will combine detailed spatial statistical methods and spatial dynamic models together with spatially resolved sociodemographic, environmental, epidemiological, and/or genetic data to disentangle the collective dynamics of infectious disease transmission to guide public health policy [5, 6]. Additionally, it will examine the spatial distribution of infectious disease burden, including the identification of hotspots and case clustering [6–9], calibrate dynamic models for forecasting the trajectory of epidemics [5, 6, 9], and simulate scenario analyses to evaluate the impact of different control strategies on epidemic control at various spatial scales [10].

## Diverse spatial approaches for infectious disease epidemiology research

**Fig. 1:**
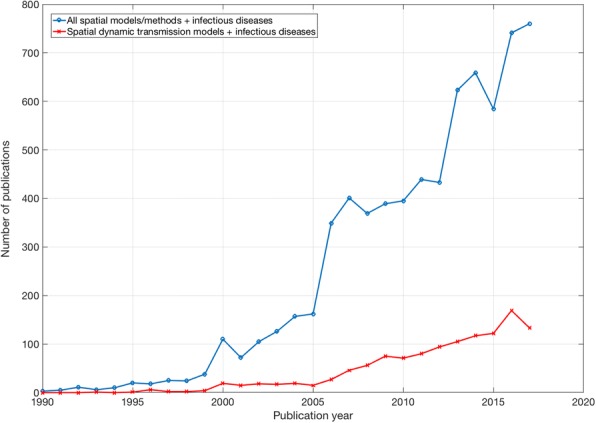
Growth in spatial modeling, 1990–2017 (Web of Science). *Search keywords for spatial modeling publications*: (spatial model AND infectious diseases) OR (spatial method AND infectious diseases) OR (agent-based model AND infectious diseases) OR (individual-based model AND infectious diseases) OR (metapopulation model AND infectious diseases) OR (microsimulation model AND infectious diseases). *Spatial dynamic transmission modeling publications*: (microsimulation model AND infectious diseases) OR (agent-based model AND infectious diseases) OR (agent-based modeling AND infectious diseases) OR (individual-based model AND infectious diseases) OR (metapopulation model AND infectious diseases) OR (metapopulation modeling AND infectious diseases)

Quantitative infectious disease investigations involving spatiotemporal data rely on two broad classes of research methods, namely spatial statistical modeling methods [11–15] and spatial transmission dynamic modeling approaches [16–21]. The application of these methodological approaches for infectious disease research has rapidly increased over the last two decades, along with major advances in computational power and an increasing amount and diversity of epidemiological and genetic data with spatial and temporal information (Fig. 1). For instance, spatial statistical methods are frequently used to uncover relationships between spatiotemporal infectious disease patterns and host or environmental characteristics [11], generate detailed maps to visualize the distribution of infectious disease morbidity or mortality [22–24], and identify hotspots or clusters [12, 13].

In this collection, Warren et al. [7] employed hierarchical Bayesian statistical modeling and epidemiological and genetic data prospectively collected from neighborhoods surrounding a prison in Lima, Peru, to assess spillover of multi-drug resistant tuberculosis (MDR-TB) strains into the surrounding community. Their findings revealed a potential spillover of TB from the prison with a radius estimated at 5.47 km and found that nine MDR-TB samples from non-inmate patients had genetic matches with genetic samples from inmate patients, suggesting that interventions targeting the prison could have benefits for the surrounding community. Moreover, they found a total of eight spatially aggregated genetic clusters of MDR-TB, of which four were within the spillover region. In another contribution, Kang et al. [8] conducted Bayesian spatiotemporal modeling to generate high-resolution maps of malaria prevalence in Madagascar from 2011 to 2016. Their findings indicate substantial changes in malaria activity over the study period and underscore the importance of monitoring spatiotemporal changes to guide control programs.

Using mathematical and statistical approaches to forecast the course of epidemics at different spatial scales in order to guide interventions is a challenging research area that has received increasing attention over the last decade [25–28]. In *BMC Medicine*, Chen et al. [9] developed a statistical framework based on LASSO regression and various spatiotemporal datasets, including weekly surveillance epidemiological data, cell phone network data, building characteristics, meteorological data, vegetation index, and public transport history data, to forecast the trajectory of dengue epidemics at the neighborhood level from 2010 to 2016 in Singapore. Their relatively simple statistical forecasting tool performed remarkably well at generating short-term forecasts (5–12 weeks ahead) with direct public health implications. An open question is whether mechanistic (based on spatial dynamic transmission models) or hybrid approaches could outperform statistical model-based forecasts such as theirs.

Applications of spatial transmission dynamic modeling approaches to investigate infectious disease transmission and control has increased over the last two decades, with a research production of less than five articles per year in 1997 to more than 120 articles per year (Fig. 1). System dynamic models have been most useful in generating scenario analyses of the potential course and severity of infectious disease epidemics [16–18, 21, 28–30], characterizing and forecasting the spatiotemporal transmission patterns of epidemic outbreaks, or assessing the effectiveness of interventions and the feasibility of achieving elimination targets. In these models, researchers artfully integrate key epidemiological characteristics of the disease and strive to capture relevant mechanisms of disease transmission, including the potential influence of environmental factors. In metapopulation models, a particular type of spatial dynamic model, the population is divided in a set of interacting population groups defined according to spatial or demographic information [16, 17, 31–33]. In comparison, agent-based or microsimulation models consider discrete individuals and model specific individual-level interactions and daily activity patterns, allowing substantial heterogeneity to be included in the population (e.g., vaccination status, healthcare worker status) [34–36].

Also in this special collection, two studies employed spatial dynamic modeling frameworks at different spatial scales to investigate the spatiotemporal dynamics and control of two mosquito-borne infectious diseases of major international concern. O’Reilly et al. [6] developed a deterministic metapopulation model where city-level populations interact according to several scenarios based on gravity and radiation mobility models and flight data to assess the spread of Zika across 90 major cities in Latin America. Their model was calibrated using epidemiological time-series data to estimate transmission parameters and project incidence in 2018. Their findings suggest that population herd immunity has been achieved, with low incidence expected in 2018. This is in line with prior simulations of Zika incidence in the Americas through February 2017 that used an agent-based model to characterize transmission based on temperature, socioeconomic and vector density [36]. Moore et al. [5] adapted an agent-based model of dengue transmission dynamics to explore the role of spatial scale ‘mismatching’ in the dynamics of the 2014–2015 chikungunya epidemic in Colombia. By calibrating models with increasingly higher spatial resolution (national, department, municipality) to national and department-level incidence data, the study demonstrates the importance of designing models that incorporate spatially resolved patterns in mosquito abundance modulated by climatic factors, population density, and movement patterns. This work echoes the spatial dynamics of other infectious diseases such as influenza in the United States [37], the Western African Ebola epidemic [28], and Zika in the Americas [36], and underscores the impact of spatial structure (metapopulation, static vs. dynamic network-based models) on disease dynamics.

## Conclusions

**Fig. 2:**
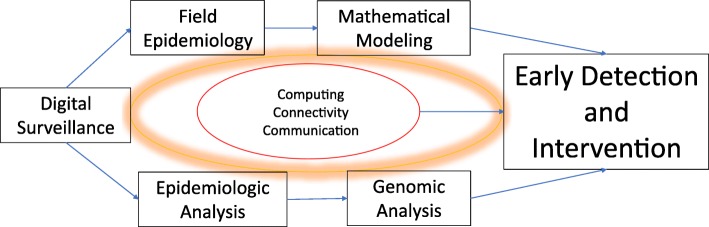
On the cusp of intervention. We are close to a fully integrated approach for early epidemic detection and intervention as evidenced by the burgeoning output of data-driven spatial modeling. Recent events, such as MERS, SARS, Ebola, and influenza, have highlighted the need for coordinated, interactive, and multidisciplinary methods and permit rapid and real-time evaluation and action

These contributions, exemplars of the burgeoning output of data-driven spatial modeling, suggest that we are close to a fully integrated approach for early epidemic detection and intervention (Fig. 2). Recent events, such as MERS, SARS, Ebola, and influenza, have highlighted the need for coordinated, interactive, multidisciplinary methods that permit rapid, real-time evaluation and public health action. Virtually all of the elements are in place, although at different stages of development. Digital surveillance is in its infancy, yet it is a rapidly developing research area [38, 39], and field epidemiology and analytical tools are well developed. Mathematical modeling, as evidenced by the contributions in this collection, as well as genomic analysis are rapidly expanding [40]. With the aid of high capacity/high speed computers, accessible from anywhere, these elements speak a mutually comprehensible language. Consider a future disease, X, whose first cases, reported by high speed communication, evoke a coordinated effort from the host country and contributors, immediate on-the-ground epidemiologic characterization, analysis to determine routes of spread, incubation periods, and infectivity, specimens to characterize the infecting agent at the molecular level (immediate vaccine development in a separate path), and models to predict spatial spread. One can envision intervention plans and implementation within (possibly) a matter of days. This description is not far from the actuality of recent events, albeit ignoring the confusion and uncertainty that would be generated by a new, as yet unexplained event, which is critical to achieving a timely and effective intervention. Those first few hours and days are crucial for political interaction and social organization to enhance or undermine the effort. Unfortunately, the latter is often the case, with issues of resource allocation and agency authority hindering rapid action. Smooth political and social coordination are every bit as important as key technical tools such as genomic technology.
